# Four thiol-oxidoreductases involved in the formation of disulphide bonds in the *Streptomyces lividans* TK21 secretory proteins

**DOI:** 10.1186/s12934-019-1175-0

**Published:** 2019-07-25

**Authors:** Sonia Gullón, Silvia Marín, Rafael P. Mellado

**Affiliations:** Departamento de Biotecnología Microbiana, Centro Nacional de Biotecnología (CNB-CSIC), c/Darwin 3, 28049 Madrid, Spain

**Keywords:** *Streptomyces lividans*, Disulphide bonds, Thiol-disulphide oxidoreductases

## Abstract

**Background:**

Bacterial secretory proteins often require the formation of disulphide bonds outside the cell to acquire an active conformation. Thiol-disulphide oxidoreductases are enzymes that catalyse the formation of disulphide bonds. The bacterium *Streptomyces lividans* is a well-known host for the efficient secretion of overproduced homologous and heterologous secretory proteins of industrial application. Therefore, the correct conformation of these extracellular proteins is of great importance when engineering that overproduction.

**Results:**

We have identified four acting thiol-disulphide oxidoreductases (TDORs) in *S. lividans* TK21, mutants in all TDOR candidates affect the secretion and activity of the Sec-dependent alpha-amylase, which contains several disulphide bonds, but the effect was more drastic in the case of the Sli-DsbA deficient strain. Thus, the four TDOR are required to obtain active alpha-amylase. Additionally, only mutations in Sli-DsbA and Sli-DsbB affect the secretion and activity of the Tat-dependent agarase, which does not form a disulphide bond, when it is overproduced. This suggests a possible role of the oxidised Sli-DsbA as a chaperone in the production of active agarase.

**Conclusions:**

Enzymes involved in the production of the extracellular mature active proteins are not fully characterised yet in *Streptomyces lividans.* Our results suggest that the role of thiol-disulphide oxidoreductases must be considered when engineering *Streptomyces* strains for the overproduction of homologous or heterologous secretory proteins of industrial application, irrespective of their secretion route, in order to obtain active, correctly folded proteins.

**Electronic supplementary material:**

The online version of this article (10.1186/s12934-019-1175-0) contains supplementary material, which is available to authorized users.

## Background

The formation of disulphide bonds is necessary for the secretory proteins in order to procure their correct function and improve their extracellular stability [1]. The disulphide bonds are formed when the thiol groups of two cysteine residues are oxidised by the action of specialized thiol-disulphide oxidoreductase proteins (TDORs). The disulphide oxidoreductases belong to the thioredoxin superfamily that contain a conserved sequence motif (CXXC) at their active sites located in a highly-conserved folding [1].

The action of an enzyme that oxidises substrate cysteines is required for the formation of disulphide bonds in the secreted proteins and, to maintain this oxidised state, that enzyme needs to be subsequently re-oxidised by other oxidase, which must transfer the accepted electrons to the last electron acceptor, usually the electron transport components [2, 3].

In the gram-negative bacteria *E. coli* five thiol-disulphide oxidoreductases have been described to be involved in disulphide bond formation in the secreted proteins. DsbA is a monomeric periplasmic enzyme that catalyses disulphide bond formation in secreted proteins immediately after their translocation into the periplasm, and it is maintained in its oxidised active state by the transmembrane protein DsbB. Proteins DsbC, a dimeric periplasmic protein, and DsbG reorganise the wrongly made non-native disulphide bonds, and both are kept in a reduced state by the membrane reductase DsbD [4, 5]. DsbD also transfer electrons to CcmG, a thioredoxin-like protein involved in the cytochrome c maturation [6]. Additionally, DsbA, DsbC and DsbG also display a chaperone activity [7–9].

In the gram-positive bacteria *Bacillus subtilis*, where the periplasm compartment is absent, the candidates for the formation of disulphide bonds in the secreted proteins appear to be BdbD acting as a thiol oxidase which is re-oxidised by BdbB and BdbC, where BdbC plays the main role. In *B. subtilis* other TDORs have been described, such as CcdA that interacts with ResA (to maintain the heme-binding site of the apocytochrome c in a reduced state) or StoA (involved in the formation of the spore cortex) additionally, YneN appears to take part in the reductive process but its function has not been determined yet [10].

*Streptomyces lividans* is a well-known gram-positive bacterium, and an efficient secretor of proteins with industrial application [11]. Up to 72% of the identified secreted proteins in *Streptomyces lividans* TK21 secretome contained two or more cysteines [12], thus suggesting the need of an efficient TDOR system. The identification and characterization of the *S. lividans* thiol-oxidoreductases involved in the secretory proteins disulphide bond formation will provide useful information to improve the use of *S. lividans* TK21 as cell factory for extracellular proteins of industrial or pharmacological interest. This work describes the identification of four thiol-disulphide oxidoreductases in *Streptomyces lividans* TK21 involved in formation of disulphide bonds in the secreted proteins.

## Results

### Identification of the thiol-disulphide oxidoreductases in *S. lividans* TK21

Using the *Bacillus subtilis* BdbD sequence to search for homologous proteins, four possible thiol-disulphide oxidoreductases were identified in the *S. lividans* TK24 genome [13] namely SLIV_08650, SLIV_27370, SLIV_27535, and SLIV_28015 containing 25%, 25%, 26% and 26% of identical residues and 45%, 41%, 45%, 43% of equivalent similar amino acids, respectively. The four candidates have the conserved Cys-X-X-Cys motif present in the thiol-disulphide oxidoreductases.

Based on the four possible candidates identified in *S. lividans* TK24, oligonucleotides containing possible regulatory region and the coding sequences of the different genes encoding the potential oxidoreductases were designed and used to amplify the corresponding genes from the *Streptomyces lividans* TK21 genome (Oxidoreductases genes, Additional file 1).

The obtained DNA fragments were subsequently sequenced and aligned against their potential gene to confirm that the obtained sequences coincide with those of the *S. lividans* TK24 genome. The candidate genes to encode the thiol-disulphide oxidoreductases in *S. lividans* TK21were named according to its localization and the similarity to the *E. coli* TDOR encoding genes *sli*-*dsbA* (*SLIV_28015* homologue), *sli*-*dsbB* (*SLIV_27535* homologue), *sli*-*dsbC* (*SLIV_08650* homologue) and *sli*-*dsbD* (*SLIV_27370* homologue).

Protein Sli-DsbA is a TDOR bacterial lipoprotein according to the SignalP server [14] and LipoP server [15, 16] that predict a lipoprotein signal peptide cleavage site between residues A24 and C25. Sli-DsbA has some residues in this sequence that appears to be important for the *E. coli* equivalent enzyme, in particular the Val-Pro residues [17] (see Additional file 2).

Protein Sli-DsbB is a membrane protein with a transmembrane domain between residues 50–72 as suggested by TMHMM server [18] that contains four cysteines localised outside the cytoplasm membrane, similar to the four essential cysteines, present in the *E. coli* DsbB, which appear in the first and second periplasmic loops [19]. Additionally, there is an arginine residue close to the cysteines residues present on the Sli-DsbB active site, as it occurs in the *E. coli* DsbB, where the arginine residue appears to be important for the transfer of electrons from DsbB to quinones in the respiratory chain [17] (see Additional file 2).

Protein Sli-DsbC is likely a secreted TDOR according to the SignalP server, that predicts the presence of a signal peptide cleavage site between residues A45 and L46. The Sli-DsbC coding sequence apparently forms a transcriptional unit with a downstream one, where the stop codon of *sli*-*dsbC* overlaps with the start codon of a gene involved in the cytochrome c-type biogenesis and which shares homology with the *B. subtilis* CcdA, an integral membrane protein functionally related to *E. coli* DsbD [20]. Protein Sli-DsbC is a secreted protein partially homologous to the *E. coli* DsbC and DsbG (see Additional file 2).

Protein Sli-DsbD is a membrane protein with a transmembrane domain between residues 34–56 as suggested by the TMHMM server, and is partially homologous to the *E. coli* DsbD and the *B. subtilis* BdbD proteins (see Additional file 2).

### Thiol-oxidoreductases involved in the formation of the disulphide bonds in the *S. lividans* strains overproducing the Sec-dependent secretory protein alpha-amylase

Individual mutant strains were constructed for each of the genes encoding the four possible TDOR candidates, as indicated in “Methods”. All mutant strains were viable and the different mutations apparently did not affect the rate of growth in liquid medium, but the Sli-DsbD deficient strain showed a delayed growth producing small colonies in R5 solid medium when compared to the wild type (not shown).

**Fig. 1:**
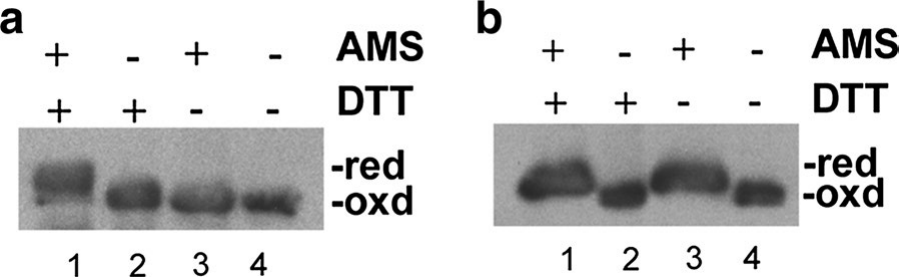
Disulphide bond formation in the model proteins. AMS alkylation and Western blot analysis with antibodies raised against AmlB (**a**) and DagA (**b**) were performed to determine the presence of disulphide bonds in the proteins studied. Samples from supernatants of overproducing alpha-amylase and agarase strains (**a**, **b**) were treated with the alkylated agent AMS (+) (lane 1 and 3) or not (−) (lane 2 and 4) and with the reducing agent DTT (+) (lane 1 and 2) or not (−) (lane 3 and 4). AMS reacts with the free thiol groups, so retarded mobility in the alkylated proteins indicated presence of reduced thiol groups. Samples not incubated with AMS were used as non-alkylated controls. The mobility of the reduced (red) and oxidised (oxd) forms of the model proteins is indicated

To study the role of the TDOR candidates involved in the formation of disulphide bonds in the *S. lividans* extracellular proteins, we have used the Sec-dependent secretory model protein alpha-amylase [21]. Mature alpha-amylase contains 10 cysteines and, consequently, has the capacity to form up to five potential disulphide bonds according to the DIANNA 1.1 web server [22]. To study the presence of disulphide bonds in alpha-amylase we incubated the thiol-alkylating reagent 4-acetamido-4′-maleimidylstilbene-2,2′-disulphonic acid (AMS) with the culture supernatant of the overproducing alpha-amylase strain with or without the reducing reagent DTT. AMS has a molecular mass of 500 Da and reacts with the free thiol groups increasing the molecular mass of the protein that can be monitored under non-reducing SDS-PAGE. The disulphide bonds are formed when the thiol groups of two cysteine residues are oxidised, therefore, proteins that form disulphide bonds do not react with AMS and their migration is not retarded. Alpha-amylase was retarded in samples treated with DTT and AMS (Fig. 1a, lane 1) but not in samples treated only with AMS (Fig. 1a, lane 3) showing that alpha-amylase has disulphide bonds as there is no reaction with AMS. Non-alkylated samples had not a retardation in the migration (Fig. 1a, lane 2 and 4).

**Fig. 2:**
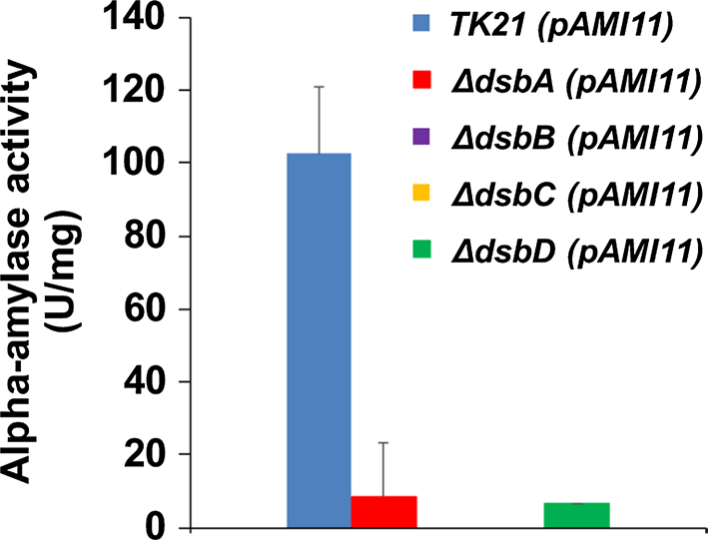
Alpha-amylase extracellular activity in the Sli-Dsbs deficient strains. Extracellular alpha amylase activity is expressed as units per mg of protein. For comparative purposes, only the alpha-amylase activity at the time of growth where the enzyme reaches its maximum extracellular presence is depicted. The data are the average of at least three independent determinations. Bars show standard error

**Fig. 3:**
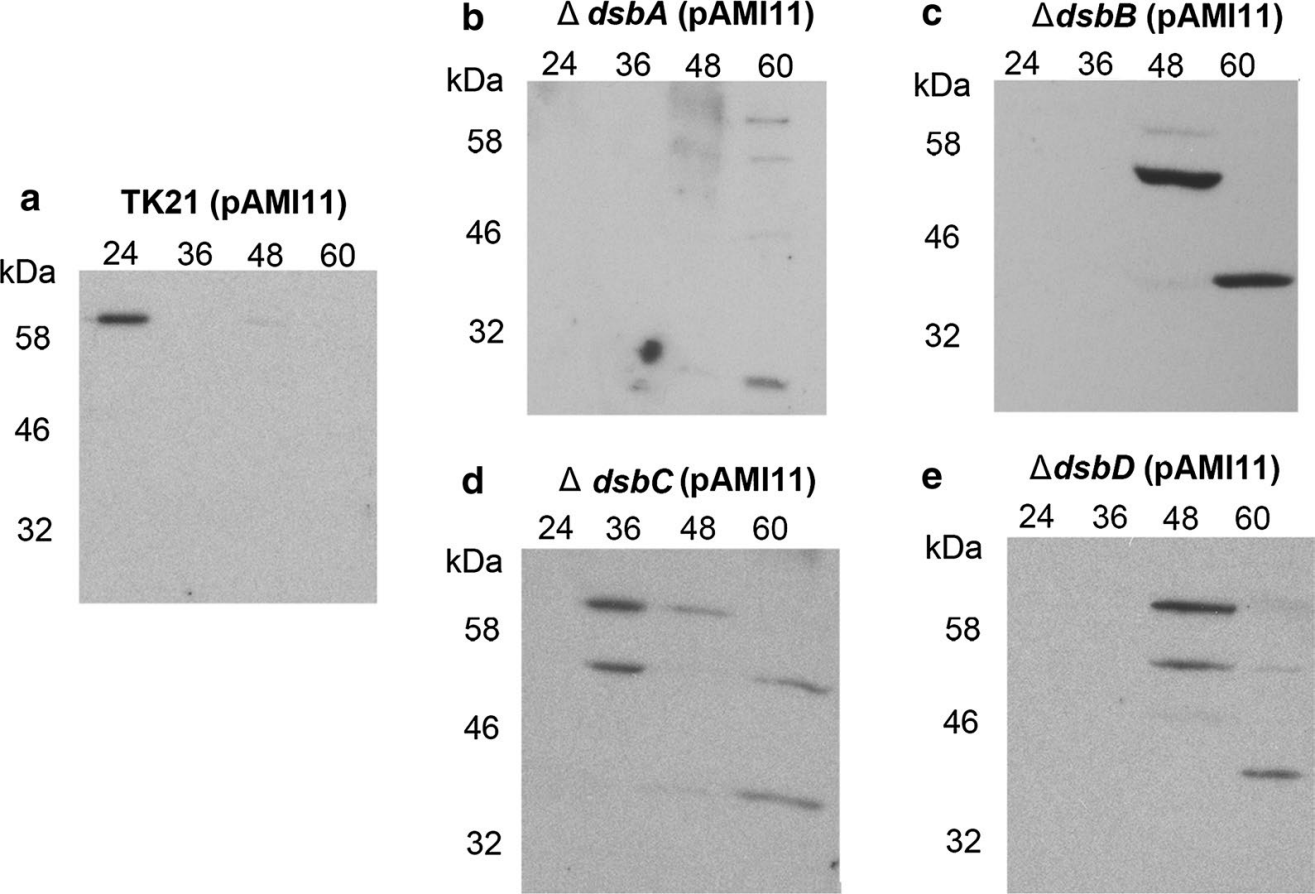
Alpha-amylase secretion pattern in the Sli-Dsbs deficient strains. Extracellular alpha-amylase present in the *S. lividans* TK21 (pAMI11) (**a**), *S*. *lividans* Δ*dsbA* (pAMI11) (**b**), *S. lividans* Δ*dsbB* (pAMI11) (**c**), *S. lividans* Δ*dsbC* (pAMI11) (**d**) and *S. lividans* Δ*dsbD* (pAMI11) at different times of growth were analysed by Western blotting with antibodies raised against AmlB

In the *S. lividans* pAMI11 strain overproducing alpha-amylase, the maximal secretion level and extracellular activity of the enzyme was observed at the late exponential phase of growth (24 h; [21]). The multicopy plasmid pAMI11, harbouring alpha-amylase, was propagated in the different Sli-Dsb deficient strains and the secretion pattern and activity of the alpha-amylase was monitored in each case (Figs. 2, 3). Alpha-amylase activity was severely reduced in all mutant strains when compared to that of the isogenic strain (Fig. 2). When the alpha-amylase secretion pattern of the deficient strains was compared to that of the isogenic strain, bands of smaller size than that of the mature alpha-amylase appeared later in growth in the respective culture supernatants (Fig. 3). The absence of any of the thiol-disulphide isomerases likely triggers accumulation of misfolded alpha-amylase, which induces the secretion stress responses, increasing the level of expression of the *cssRS* two-component operon, which, in turn, regulates positively the expression of the three specific genes encoding the HtrA-like proteases responsible for the extracellular degradation of misfolded proteins [23, 24].

**Fig. 4:**
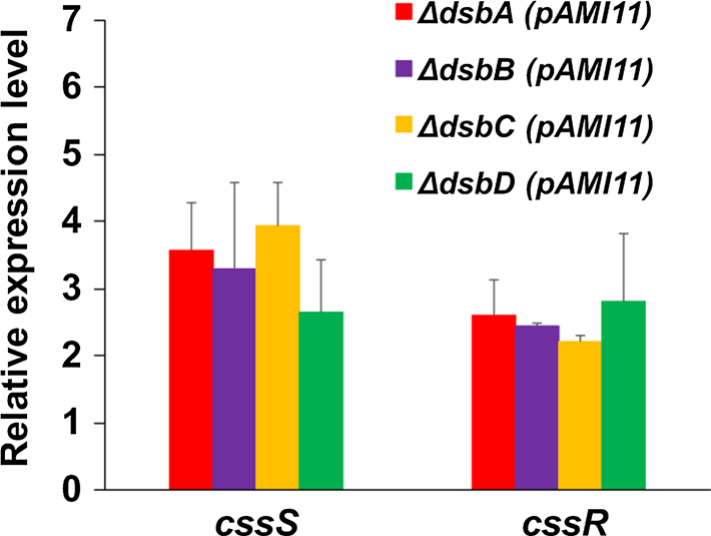
Induction of the secretion stress response in the Sli-Dsbs deficient strains overproducing alpha-amylase. Quantitative RT-PCR analyses showed that the expression of the *cssS* and *cssR* increased in the Sli-Dsbs deficient strains overproducing alpha-amylase with respect to the wild type strain overproducing alpha-amylase. The values are the mean of at least three biological replicates. Bars show the standard error

Therefore, we have analysed if, when overproducing alpha-amylase, mutations in each of the thiol-disulphide oxidoreductases, increase the level of expression of the *cssRS* two-component system in comparison to that of the wild type at the late exponential phase of growth (24 h), as determined by qRT-PCR analyses showing that the secretion stress response increases (Fig. 4).

Transcriptional analyses revealed that the expression level of *sli*-*dsbC* and *sli*-*dsbD* increased at 24 h of growth in the *S. lividans* TK21pAMI11 alpha-amylase overproducer strain when compared with the strain that carries plasmid pIJ487 not containing the alpha-amylase gene (*sli*-*dsbC*: 9.97 ± 0.32; *sli*-*dsbD*: 3.87 ± 1.88), whereas the expression level of *sli*-*dsbA* and *sli*-*dsbB* remains unaltered at different times of growth.

### Thiol-oxidoreductases are required to obtain an active protein in the *S. lividans* strain strains overproducing the Tat-dependent secretory protein agarase

Up to 48% of the *S. coelicolor* Tat secreted proteins [25] have not cysteine residues or have only one cysteine residue, while the rest have the possibility to form disulphide bonds. Agarase is a *S. coelicolor* Tat protein and the mature enzyme contains two cysteines, a feature not shared with other β-agarases [26]. Nevertheless, agarase was retarded in samples from the agarase overproducer strain both treated with DTT and AMS (Fig. 1b, lane 1), and only with AMS (Fig. 1b, lane 3), showing that agarase does not form a disulphide bond. Non-alkylated samples were not retarded in their migration (Fig. 1b, lane 2 and 4).

The multicopy plasmid pAGAs5 [27] harbouring the *S. coelicolor dagA* gene encoding agarase was propagated in the different Sli-Dsb deficient strains. No effect on the growth rate was observed when the deficient strains were compared with the isogenic one.

**Fig. 5:**
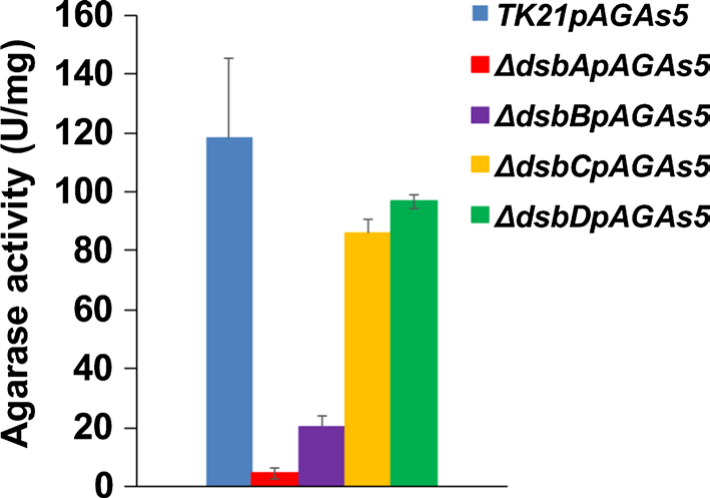
Agarase extracellular activity in the Sli-Dsbs deficient strains. Agarase in the culture supernatants is expressed as units per mg of dry weight. For comparative purposes, only the alpha-amylase activity at the time of growth where the enzyme reaches its maximum extracellular presence is depicted. The data are the average of at least three independent determinations. Bars show standard error

**Fig. 6:**
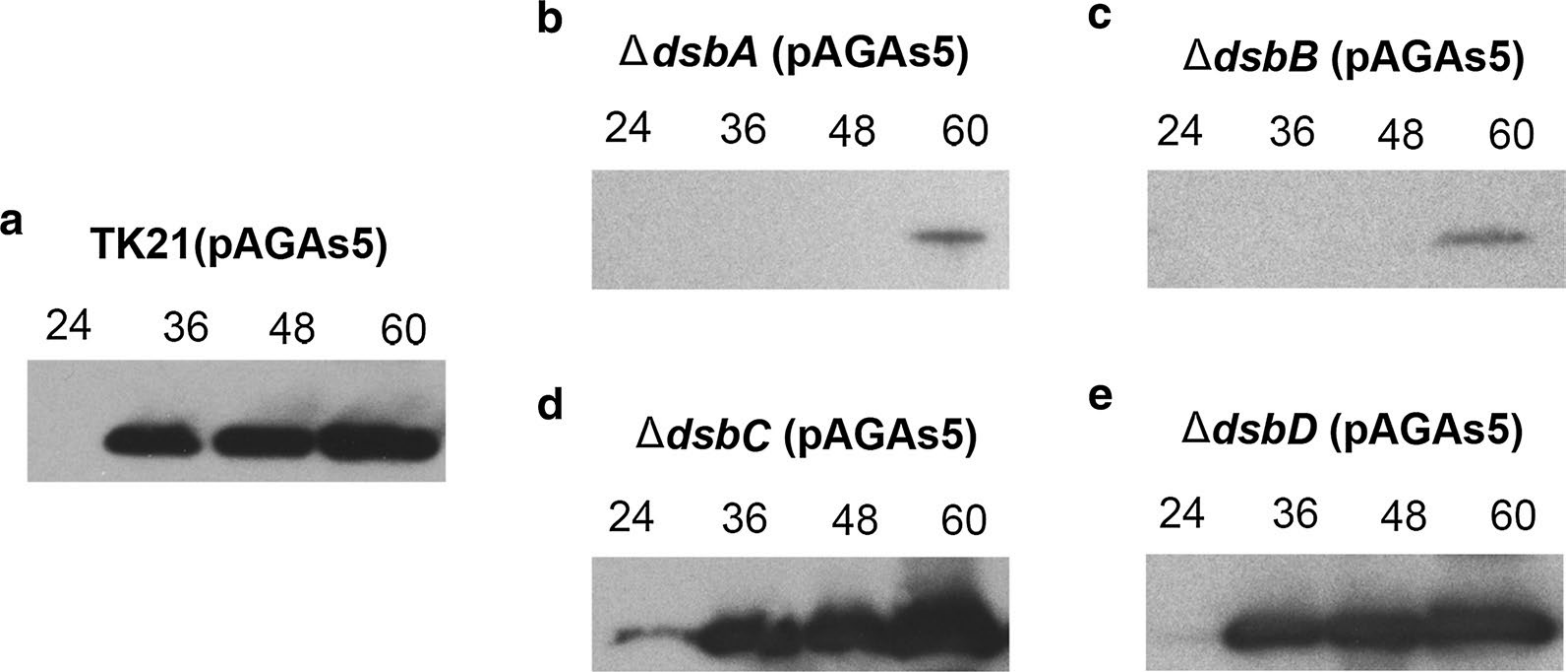
Agarase secretion pattern in the Sli-Dsbs deficient strains. Extracellular agarase present in the *S. lividans* TK21 (pAGAs5) (**a**), *S*. *lividans* Δ*dsbA* (pAGAs5) (**b**), *S. lividans* Δ*dsbB* (pAGAs5) (**c**), *S. lividans* Δ*dsbC* (pAGAs5) (**d**) and *S. lividans* Δ*dsbD* (pAGAs5) at different times of growth were analysed by Western blotting with antibodies raised against DagA

The production of mature agarase in *S. lividans* TK21pAGAs5 reached its maximum level at the stationary phase [21]. Surprisingly, the agarase secretion pattern and the extracellular activity appear severely affected in the Sli-DsbA and Sli-DsbB deficient strains, strongly suggesting that the mature agarase needs the action of these thiol-disulphide oxidoreductases to acquire its extracellular, correctly folded, active configuration (Figs. 5, 6). The Sli-DsbC and Sli-DsbD deficiencies seem to cause no effect to the agarase extracellular levels or the enzyme activity at the stationary phase (Figs. 5, 6). This suggests that Sli-DsbA and Sli-DsbB are needed for the activity and extracellular stability of the mature agarase. Transcriptional analyses revealed that the expression levels of *sli*-*dsbA, sli*-*dsbB, sli*-*dsbC* and *sli*-*dsbD* remain unaltered in *S. lividans* pAGAs5 at the different times of growth when compared with the *S. lividans* pIJ487 strain (not shown).

## Discussion

The genome of the gram-positive bacterium *Streptomyces lividans* encodes a considerable amount of secretory proteins of industrial interest. Most of these contain two or more cysteine residues in their primary structure, and the action of specific thiol-disulphide oxidoreductases is needed to form the correct disulphide bonds, thus ensuring both, stability and proper functioning of the extracellular proteins.

The formation of the disulphide bond requires the extracellular oxidation of the secreted protein by a thiol-disulphide oxidoreductase; this oxidation requires in turn the action of another membrane anchored thiol-disulphide oxidoreductase to re-oxidase the already reduced, first acting, oxidoreductase, so that oxidation–reduction process could be kept working effectively.

The construction of disulphide bonds is concomitant with the translocation and extracellular appearance of the secreted protein; therefore, it is not infrequent that the disulphide bond formation could take place between cysteine residues close enough to one another in the primary structure of the secreted protein. When the protein contains more than two cysteine residues, wrongly formed disulphide bonds need to be corrected by the action of a specialised thiol-disulphide oxidoreductase, favouring the acquisition of the properly folded mature protein structure [1].

There is little information about the disulphide bond formation in *Streptomyces*. We have identified four thiol-disulphide oxidoreductases (TDORs) involved in the formation of the disulphide bonds in *S. lividans* TK21 secretory proteins. Two of these enzymes (Sli-DsbB and Sli-DsbD) reside in the membrane, whereas Sli-DsbC and the lipoprotein Sli-DsbA act extracellularly. The *sli*-*dsbA* gene expression results negatively regulated in a *S. lividans* TK21 strain defective in the type II signal peptidase Lsp [28], which qualifies Sli-DsbA as lipoprotein.

Individual mutants deficient in each of the TDORs were made, and their effect in the activity and extracellular stability of the overproduced Sec-dependent model protein alpha-amylase containing disulphide bonds (Fig. 1a), and the Tat-dependent model protein agarase that does not form a disulphide bond (Fig. 1b), were analysed.

The four oxidoreductases seem to be needed to obtain an active and stable alpha-amylase (Figs. 2, 3) as the secretion pattern of this enzyme appears to match the observed one when misfolded alpha-amylase accumulates in the supernatant [23, 24]; being the effect more severe in the case of the Sli-DsbA deficient strain. The alpha-amylase extracellular activity was basically non-existent in all mutants. The accumulation of extracellular misfolded alpha-amylase triggers the expression of the CssRS two-component system at the late exponential phase of growth; which in turn induces the synthesis of the three HtrA-like proteases that degrade the incorrectly folded alpha-amylase [24]. The expression of this two-component system is induced in all the thiol-disulphide oxidoreductase deficient strains that overproduce alpha-amylase at the late exponential phase of growth (24 h) (Fig. 4).

Despite agarase lacking disulphide bonds, the agarase secretion pattern and agarase activity were affected in the Sli-DsbA and Sli-DsbB deficient strains overproducing agarase (Figs. 5, 6). No deficiencies on the stability or activity of the secreted agarase were detected when the enzyme was overproduced in the Sli-DsbC or Sli-DsbD deficient strains (Figs. 5, 6).

Previously, it has been described that DsbA has a chaperone activity in enzymes devoid of disulphide bonds [7], suggesting that Sli-DsbA could act as a chaperone for the folding of the protein agarase (Fig. 1b). Secretory proteins using the Tat route are thought to appear fully folded outside the bacterial cell [29] and up to 27 proteins have been experimentally confirmed to use the Tat pathway in *S. coelicolor* [25]. It appears that when agarase is overproduced in the absence of Sli-DsbA, the folding capacity of the Tat translocase system may not work to its full capacity, which opens the intriguing possibility that, despite the general believe that Tat secreted proteins are fully folded, actually, proper Tat secretion may require, at least in some cases, such as that of agarase, a combination of two concomitantly occurring processes: an efficient translocation combined with the equally efficient action of the DsbA lipoprotein, that should reside in close proximity to the Tat translocase. Further experimental work is clearly needed to characterize their relationship.

The deficiencies on the stability and secretion of the secreted agarase in the Sli-DsbB deficient strain indicate that Sli-DsbB could be the candidate to maintain Sli-DsbA in an oxidised state, as has been described for the formation of disulphide bond by *E. coli* DsbA, but also it appears that oxidised DsbA is a strong promoter of folding as suggested in *E. coli* [30], and similar to other chaperones [31]. Nevertheless, the chaperone activity in the *E. coli* DsbC and DsbG is not dependent on the redox status of their cysteines [9, 32].

Alpha-amylase secretion was severely affected in the Sli-DsbA deficient strain, while in the rest of the mutants the accumulation of misfolded alpha-amylase was observed (Fig. 3; [23, 24]), suggesting a main role in the formation of the disulphide bond for Sli-DsbA. Thereby, once the protein has been translocated outside the cytoplasmic membrane, Sli-DsbA would oxidise the emerging cysteines catalysing the disulphide bond formation. Alpha-amylase contains five potential disulphide bonds suggesting the need to have the proof-reader thiol-disulphide oxidoreductases closely located to correct wrong disulphide bonds and render a fully functional protein. Sli-DsbC would be the candidate to reconstruct incorrect alpha-amylase disulphide bonds, and Sli-DsbD would restore the Sli-DsbC active state, suggesting that Sli-DsbC and Sli-DbD form a pair, in an scenario similar to that described for *E. coli* Dsbs [33]. Additional experiments would be desirable to ascertain the extent to which this model can be generalized to other secretory proteins containing disulphide bonds.

Whereas the obtained results may permit to predict the potential primary function of the two-extracellular acting Dsbs (Sli-DsbA-DsbC), it may not be the case for the two Dsbs resident in the membrane (Sli-DsbB-DsbD). Although Sli-DsbB seems to form a pair with Sli-DsbA in the case of agarase, we cannot predict whether this will be the same in the alpha-amylase case where the four Sli-Dsbs seem to be needed to obtain an extracellularly active protein.

The thiol-oxidoreductases have their cysteine residues oxidised so that they can be reduced when the enzyme oxidises the target protein, and the now reduced oxidoreductases need to be oxidised again. This oxidation normally is obtained in bacteria via the subsequent reduction of proteins from the oxidative phosphorylation pathways [2, 3]. It would not be surprising if in a way or another, as it occurs in *E. coli* [34] Sli-DsbC and Sli-DsbD may play a role in the transport of thioredoxin reducing power to extracellular targets in *Streptomyces*.

Equally interesting may be to investigate if the overproduction of thiol-disulphide oxidoreductases may increase the extracellular stability and activity of the two model secretory proteins, alpha amylase in particular; although, as somehow expected, preliminary attempts to propagate in high copy number any of the genes encoding the identified four oxidoreductases have proven not to render any viable bacterial strains.

## Conclusions

The obtained results indicate that regardless of the route used by the over synthesised secretory proteins in *S. lividans*, the role played by the thiol-disulphide oxidoreductases procuring them a correctly folded structure have to be taken into account, in type and relative quantities, when engineering *Streptomyces* strains for homologous or heterologous secretory protein overproduction.

## Methods

### Bacterial strains, plasmids and media

The *S. lividans* TK21 wild-type strain [35] and its derivatives were cultured in liquid NMMP medium using mannitol as carbon source [36], apramycin (50 μg/ml), thiostrepton (50 μg/ml), kanamycin (50 μg/ml) and chloramphenicol (25 μg/ml) were added to the R5 and MS solid media, when required.

### Bioinformatic programs

SignalP 5.0 server [14] and LipoP 1.0 server [15, 16] were used to predict the presence of the signal peptide and lipoprotein signal peptide in the protein sequence analysed. TMHMM server v.2.0 [18] was used to predict the presence of transmembrane domains. The presence of disulphide bonds in the proteins was predicted using DIANNA 1.1 web server [22]. The multiple sequence alignment was performed using default settings within the ClustalW [37].

### Construction of gene disrupted mutants

To construct the *sli*-*dsbA*, *sli*-*dsbB*, *sli*-*dsbC* and *sli*-*dsbD* mutant strains, oligonucleotides 1940dis_Fw and 1940 dis_Rv, 2035dis_Fw and 2035dis_Rv, 5993FPAC2 and 5993RPAC2 and 2067FPAC and 2067RPAC were used to amplify a 657, 441, 652 and 650 bp DNA fragments respectively from the *S. lividans* TK21 genome (Additional file 1 Oxidoreductases mutants).

The 441, 652 and 650 bp DNA fragments were inserted into plasmid pOJ260 [38] through its unique *Xba*I and *Pst*I sites to generate plasmids containing *sli*-*dsbB*, *sli*-*dsbC* and *sli*-*dsbD* respectively. The 657 bp DNA fragment was inserted into plasmid pO260 through its unique *Bam*HI and *Eco*RI sites to generate a plasmid containing *sli*-*dsbA*. The plasmids were used to conjugate *E. coli* to *Streptomyces*, as described [39]. *E. coli* ET12567 carrying the non-transmissible plasmid pUZ8002 was used for conjugation [40]. Apramycin resistant strains containing the disrupted genes *sli*-*dsbA, sli*-*dsbB, sli*-*dsbC and sli*-*dsbD*, were selected upon verification of the disruption by PCR amplification (not shown).

Plasmid pAMI11 [41] and plasmid pAGAs5 [27] carrying the *S. lividans* gene *amlB* and the *S. coelicolor* agarase gene *dagA* respectively, were used to transform the *S. lividans* TK21, *S. lividans* Δ*dsbA*, *S. lividans* Δ*dsbB*, *S. lividans* Δ*dsbC* and *S. lividans* Δ*dsbD* protoplasts. Plasmid pIJ487 were propagated into the *S. lividans* TK21 and thiol-disulphide oxidoreductases mutant strains to generate the corresponding isogenic strains.

### Oxidoreductases genes amplification

The oligonucleotides used to amplify the *sli*-*dsbs* genes with their respective predicted regulatory region are described in Additional file 1.

### Quantitative real time PCR (qRT-PCR)

Total RNA was isolated from bacteria growing cultures at different phases of growth (24 h, 36 h, 48 h) using the RNeasy midi Kit (Qiagen). Cell lysates were extracted twice with phenol–chloroform before being loaded onto RNeasy midi columns (Qiagen) for RNA purification. DNA potentially contaminating the RNA preparations was removed by incubation with RNase-free DNAse (Ambion) and its absence was tested by quantitative real time PCR amplification in the absence of reverse transcriptase. Complementary DNA was synthesised using the High Capacity Archive kit (Applied Biosystems). Quantitative real time PCR (qRT-PCR) was performed using SYBR Green technology in an ABI Prism 7300 Sequence Detection System (Applied Biosystems). Samples were initially denatured by heating at 95 °C for 10 min. A 40-cycle amplification and quantification program was then followed (95 °C for 15 s and 60 °C for 1 min) by a single fluorescence measurement per cycle, according to the manufacturer’s recommendations. Subsequently a final extension cycle (72 °C, 1 min) was performed. Three biological samples from the different bacterial cultures were amplified in triplicate in separate PCR reactions. All PCR products were between 50 and 150 bp in length.

A melting curve analysis was conducted after amplification to distinguish the targeted PCR products from the non-targeted ones. The melting curves were obtained by heating at temperatures ranging from 60 to 95 °C at a rate of 0.2 °C/s, with continuous fluorescence scanning. The *hrdB* transcript was carried out as an internal control to quantify the relative expression of the target genes. The *hrdB* transcript has currently been used as a reference to normalise the relative expression of *Streptomyces* genes. The oligonucleotides used as primers to amplify oxidoreductases transcripts are indicated in Additional file 1 (qRT-PCR analysis). The oligonucleotides used as primers to amplify the two-component system CssRS were described previously [23].

### Protein analysis and Western blot experiments

Supernatants from the oxidoreductases deficient strains overproducing alpha-amylase (AmlB) and agarase (DagA) grown in NMMP medium [12] were processed as described [23]. For Western blot analysis, extracellular proteins were fractionated by SDSPAGE in 10 and 12%(w/v) acrylamide gel [43].

Gel-fractionated proteins were transferred onto immobilon polyvinylidene difluoride membranes (Milipore), as described [44]. The transferred material was incubated with polyclonal antibodies raised against *S. lividans* TK21 AmlB (a gift from C. Isiegas) and *S. coelicolor* agarase (DagA; [42] followed by incubation with HRP-conjugated protein A (Invitrogen Laboratories) as described before [23].

### Enzyme activity

To determine extracellular alpha-amylase activity and agarase activity, the supernatants from the aliquots of bacterial cell cultures were concentrated at different phases of growth phases of growth by precipitation with ammonium sulphate brought to 80% saturation; the precipitated protein was collected by centrifugation at 13,000×*g* for 30 min and dissolved in 20 mM phosphate buffer (pH 7). The alpha-amylase and agarase activities were estimated by determining the amount of reducing sugars released from starch and agarose, respectively. Alpha-amylase and agarase activities were determined as previously described [23, 42].

One unit of alpha-amylase was defined as the amount of an enzyme necessary to produce reducing sugar equivalent to 1 μmol of glucose in 30 min under the assay conditions. The specific activity, measured as units per mg of protein, was the average of at least three independent determinations.

One unit of agarase activity is the amount of enzyme that increased absorbance at 450 nm by 0.001/min of incubation under the assay conditions. The specific activity was expressed as units per mg of dry weight.

### Alkylation of thiol groups in the model proteins

Supernatants from the aliquots of bacterial cell cultures at different phases of growth (24 h for the overproducer alpha-amylase strain and 60 h for the overproducer agarase strain) were treated with the alkylating reagent 4′-acetamido-4′-maleimidylstilbene-2,2′-disulphonic acid (AMS; ThermoFisher Scientific) as reported previously [45].

Four aliquots of 5 ml for the overproducer agarase strain and 15 ml for the overproducer alpha-amylase strain were taken. Two of these were incubated with dithiothreitol (DTT; 25 mM) to obtain the positive control of reduced proteins. After incubation for 10 min on 37 °C the four aliquots were precipitated with trichloroacetic acid (TCA 10% w/v) as described [23]. Two of these protein pellets, one of them previously treated with DTT, were resuspended in 75 μl of a freshly prepared alkylation buffer (150 mM Tris HCl pH 7.5, 1% w/v SDS, 5% v/v glycerol, 15 mM AMS) to obtain AMS positive samples. The other two samples were resuspended in 75 μl without AMS. After incubation for 1 h with moderate shaking at 22 ºC, 75 μl of non-reducing SDS-PAGE sample buffer was added and all samples were boiled before loading for SDS-PAGE.

## Additional files

**Additional file 1.** Primer sequences.

**Additional file 2.** Alignment of putative *S. lividans* thiol-disulfide oxidoreductases (TDOR) Representative thiol-disulfide oxidoreductases *B. subtilis* TDOR (Bs-Bdb) and *E. coli* TDOR (Ec-Dsb) were aligned using ClustalW [37] with the *S. lividans* TDOR (Sli-Dsb) using default settings. Asterisks indicated identical amino acids. A) The conserved CXXC motif are highlighted in red and the Val-Pro motif residues are highlighted in blue. B) The conserved CXXC motif and the arginine residue are highlighted in red and green, respectively. The cysteines residues localised outside of the cytoplasm membrane are highlighted in yellow. C) and D) The conserved CXXC motif are highlighted in red.

## Data Availability

All data generated and/or analysed during this study are included in this article.
