# Supplementary material for: Four thiol-oxidoreductases involved in the formation of disulphide bonds in the Streptomyces lividans TK21 secretory proteins
Source: Microb Cell Fact. 2019 Jul 25;18:126. doi: 10.1186/s12934-019-1175-0 (PMC6657201; doi:10.1186/s12934-019-1175-0)
Supplement: Supplementary file 1 — Additional file 1. Primer sequences. [file 12934_2019_1175_MOESM1_ESM.docx]

**Additional file 1. Primer sequences**

| **Primer pairs** | **Sequence (5’-3’)** | **Target fragment** | **Restriction site for cloning** | **Product length (bp)** |
| --- | --- | --- | --- | --- |
| *Oxidoreductases mutants* | |  |  |  |
| 1940dis_FW | GTTGGATCCCTACGCTTCCCTGGACGAC | *sli-dsbA* | *Bam*HI | 657 |
| 1940dis_RV | GGTGAATTCGGAGCGGTACTTCATGTCCT |  | *Eco*RI |  |
| 2035dis_FW | GGGCTGCAGGTCTGCGTGCTGGGA | *sli-dsbB* | *Pst*I | 441 |
| 2035dis_RV | GGGTCTAGAGTCGAGCAGCTTGTTCTCGT |  | *Xba*I |  |
| 2067FPAC | GGGCTGCAGAGGTCAGGCGCCAGGTCATCGTG | *sli-dsbD* | *Pst*I | 650 |
| 2067RPAC | GGGTCTAGACACCTTGTCGTTGATCTTGATGG |  | *Xba*I |  |
| 5993FPAC2 | GGGCTGCAGACGGTCGCTGCCCTGGCGGTGC | *sli-dsbC* | *Pst*I | 652 |
| 5993RPAC2 | GGGTCTAGATGGGCTGGGCGCCCAGGATCG |  | *Xba*I |  |
| *Oxidoreductases genes* | |  |  |  |
| 1940multiFW | GTGTCTAGAGTACCGCCGCAGGGCAAC | *sli-dsbA* | *Xba*I | 1131 |
| 1940multiRVA | GCGCATATGACTGCCGAGGTCGCTGTACCCAC |  | *Nde*I |  |
| 2035multiFW | GCCTCTAGAGCGTCGGCCTCGGAGTCT | *sli-dsbB* | *Xba*I | 1141 |
| 2035multiRV | CTCGGATCCAGTTCCATGGCAGGGTCG |  | *Bam*HI |  |
| 2067multiFWA | GTGTCTAGAACCACCTTCCGGCCGCTG | *sli-dsbD* | *Xba*I | 1069 |
| 2067multiRVB | GTTCATATGTCACTTGGTGACGCCCGCGTC |  | *Nde*I |  |
| 5993multiFWA | GGCTCTAGAGGTGCTCTCCTTCTTCGCCGTC | *sli-dsbC* | *Xba*I | 1159 |
| 5993multiRV | GGCCATATGAGAAGGCGGGCAGCAGCA |  | *Nde*I |  |
| *qRT-PCR analysis* | |  |  |  |
| 1940RTFWA | CGAGTACCACGAGGTGTTGT | *sli-dsbA* |  | 151 |
| 1940RTRVA | GAAGGAGCGGTACTTCATGTC |  |  |  |
| 2035RTFWA | CCAGGAGTGCGTGAAGAAC | *sli-dsbB* |  | 161 |
| 2035RTRVA | CCACCATCTGCTTTGAGCTT |  |  |  |
| 2067RTFW | CATGGAGGACGGCGACTAC | *sli-dsbD* |  | 157 |
| 2067RTRV | GGAGTACAGCGCGGTCTT |  |  |  |
| 5993RTFW | CGGTGGTGCTGATCGAGTA | *sli-dsbC* |  | 150 |
| 5993RTRV | CGGACTCCTCGCCGAAGAT |  |  |  |
