# Supplementary material for: Four thiol-oxidoreductases involved in the formation of disulphide bonds in the Streptomyces lividans TK21 secretory proteins
Source: Microb Cell Fact. 2019 Jul 25;18:126. doi: 10.1186/s12934-019-1175-0 (PMC6657201; doi:10.1186/s12934-019-1175-0)
Supplement: Supplementary file 2 — Additional file 2. Alignment of putative S. lividans thiol-disulfide oxidoreductases (TDOR) Representative thiol-disulfide oxidoreductases B. subtilis TDOR (Bs-Bdb) and E. coli TDOR (Ec-Dsb) were aligned using ClustalW [37] with the S. lividans TDOR (Sli-Dsb) using default settings. Asterisks indicated identical amino acids. A) The conserved CXXC motif are highlighted in red and the Val-Pro motif residues are highlighted in blue. B) The conserved CXXC motif and the arginine residue are highlighted in red and green, respectively. The cysteines residues localised outside of the cytoplasm membrane are highlighted in yellow. C) and D) The conserved CXXC motif are highlighted in red. [file 12934_2019_1175_MOESM2_ESM.docx]

**Additional file 2. Alignment of putative *S. lividans* thiol-disulfide oxidoreductases (TDOR)** Representative thiol-disulfide oxidoreductases *B.subtilis* TDOR (Bs-Bdb) and *E.coli* TDOR (Ec-Dsb) were aligned using ClustalW [37] with the *S. lividans* TDOR (Sli-Dsb) using default settings. Asterisks indicated identical amino acids. A) The conserved CXXC motif are highlighted in red and the Val-Pro motif residues are highlighted in blue. B) The conserved CXXC motif and the arginine residue are highlighted in red and green, respectively. The cysteines residues localised outside of the cytoplasm membrane are highlighted in yellow. C) and D) The conserved CXXC motif are highlighted in red.

A)

Sli-DsbA MGNRRRRSVASAVVMVVLSLALGACGTRAKAADADAGEAGRAGAPYASLDDAPEKLGEDG

Bs-BdbD MKKKQQSSAKFAVILTVVVVVLLAAIVIFNNKTEQGNEAVSG---------QPSIKGQP-

Ec-dsbA -----MKKIWLALAGLVLAFSASAAQYEDGKQYTTLEKPVAG------------------

. *: *: . *. :. .

Sli-DsbA TTIMVGDPDAPVTVHLYEDPR**CPVC**EEFEQRGGGPVLRDALLRGKVKTEYTLASFLDDRM

Bs-BdbD ---VLGKDDAPVTVVEFGDYK**CPSC**KVFNSDIFPKIQKDFIDKGDVKFSFVNVMFHGK--

Ec-dsbA ----------APQVLEFFSFF**CPHC**YQFEEVLHISDNVKKKLPEGVKMTKYHVNFMGGDL

. * : . ** * *:. . ** . * .

Sli-DsbA GGSGSKKAVNALRAALEAGKFTEYHEVLYDNQPEEAVDGFTDAFLLRLAGRVEGLRGPAF

Bs-BdbD -GSRLAALASEEVWKEDPDSFWAFHEKLFEKQPDTEQEWVTPAVLGDLAKSTTKIKPETL

Ec-dsbA GKDLTQAWAVAMALGVEDKVTVPLFEGVQKTQTIRSASDIRDVFIN------AGIKGEEY

. . : .* : ..*. . . ..: ::

Sli-DsbA DAAVKDMKYRSFVTASEKAYDRAGGPKEPTGPGTPTAVINDVR**VP**AEYSGLLFDTEGFTS

Bs-BdbD KDNLDKETFASQVEKDSELNQKMN------IQATPTIYVNDKVIN-----------KFMD

Ec-dsbA DAAWNSFVVKSLVAQQEKAAADVQ------LRG**VP**AMFVNGKYQLNPQGMDTSNMDVFVQ

. .. * * ..: ..*: :*. * .

Sli-DsbA LLALIAQRPQEWGEDLFT

Bs-BdbD YDEIKETIEKELKGK---

Ec-dsbA QYADTVKYLSEKK-----

.*

B)

Ec-DsbB ---------------------------------------------------MLRFLNQCS

Bs-BdbC ----------------------------------------------------MKNRIVCL

Sli-DsbB MDLGPGRRAAPPRFVLRGVSEKNRQGKRGAREKLAAEREKQRSRDKRRRALIVGASVVCV

: *

Ec-DsbB QGRGAWL---------------------------LMAFTALALELTALWFQHVMLLKP**CV**

Bs-BdbC YAS---------------------------------WVVALVAMLGSLYFSEIRKFIP**CE**

Sli-DsbB LGLAAVIGVVAANAGKDDGSESAGPVVAPSGAQGKDGLAIPVGEESAKSTLTVWEDFR**CP**

. .. . : : *

Ec-DsbB **LC**IYERCALFG--------------VLGAALIGAIAPKTPLRYVAMVIWLYSAFRGVQVT

Bs-BdbC **LC**WYQRILMYP--------------LVLLLGIATFQGDTRVKKYVLPMAIIGAF----IS

Sli-DsbB **AC**KAFELAYRNTIHELTDAGQLKVEYHLATIIDGNMGGTGSRKAANAAACAQDAGKFPPY

* . * * : .

Ec-DsbB YEHTMLQLYPSPFATCDFMARFPEWLPLDKWVPQVFVASGDCAERQWEFLGLEMPQWLLG

Bs-BdbC IMHYLEQKVPGFSGIKPCVSGVPCSGQYINWFGFITIP----------------------

Sli-DsbB HDVLYDNQPPETDDAFADENKLLDLAGKVDGLDTTLFQECVKNGKHNSWVEKSNKAFQNG

: * . . . .

Ec-DsbB IFIAYLIVAVLVVISQPFKAKKRDLFGR-------

Bs-BdbC -FLALIAFILIIIFMCLLKGEKS------------

Sli-DsbB GFSGTPTVLLDGKNIYQDRSMTPAKLKQMVEDANK

* . . : :. .

C)

Sli-DsbC -------------------------------MTGSTPSSPASPSSSRPPSASRRSRRRTV

Ec-DsbC ---------------------MKKGFMLFTLLAAFSGFAQADDAAIQQTLAKMGIKSSDI

Ec-DsbG MTVIGYAFYSTFALTEKDKLMLKKILLLALLPAIAFAEELPAPVKAIEKQGITIIKTFDA

: . . :

Sli-DsbC AALAVLAAAAVTAAFALTLDDADNREEK-------AGEPAAVTASAAPAPADEGLLALAR

Ec-DsbC QPAPVAGMKTVLTNSGVLYITDDGKHII-------QGPMYDVSGTAPVNVTNKMLLKQLN

Ec-DsbG PGGMKGYLGKYQDMGVTIYLTPDGKHAISGYMYNEKGENLSNTLIEKEIYAPAGREMWQR

*.:. * : : .

Sli-DsbC RDASDPLAIGRADAPVVLIEYSDFQ**CPFC**GRFARETKPELLRSYVDKGTLRIEWRNFPIF

Ec-DsbC ALEKEMIVYKAPQEKHVITVFTDIT**CGYC**HKLH-----EQMADYNALG-ITVRYLAFPRQ

Ec-DsbG MEQSHWLLDGKKDAPVIVYVFADPF**CPYC**KQFW-----QQARPWVDSGKVQLRTLLVGVI

.. : : :: ::* * :* :: : : * : :. .

Sli-DsbC GEESEQAALAGWAAGRQNKFWEFHDVAYGKPRERNTGAFDAENLVAMAREAGIADIERFQ

Ec-DsbC GLDSDAEKEMKAIWCAKDKNKAFDDVMAGKSVAPASCDVDIADHYALGVQLGVSGTP---

Ec-DsbG KPESPATAAAILASKDPAKTWQQYEASGGKLKLNVPANVSTEQMKVLSDNEKLMDDLG--

:* * :. ** . .. : .:. : : .

Sli-DsbC ADMASDEARGAVRADQEEGYTLGVTSTPAFLVNGRPILGAQPTDTFEEAVETAAKAAKTA

Ec-DsbC ---------------------------AVVLSNGTLVPGYQPPKEMKEFLDEHQKMTSGK

Ec-DsbG -----------------------ANVTPAIYYMSKENTLQQAVGLPDQKTLNIIMGNK--

... . *. .: .

Sli-DsbC NTTKGAGR

Ec-DsbC --------

Ec-DsbG --------

D)

Sli-DsbD ------------------------------------------------------------

Bs-BdbD ------------------------------------------------------------

Ec-DsbD MAQRIFTLILLLCSTSVFAGLFDAPGRSQFVPADQAFAFDFQQNQHDLNLTWQIKDGYYL

Sli-DsbD ----MSKRNSQAAKTAARERLRQERERQAKRDKVRRQVIVAASIVGVLAIAGGISYAVVQ

Bs-BdbD -------------------------MKKKQQSSAKFAVILTVVVVVLLAAIVIFNNKTEQ

Ec-DsbD YRKQIRITPEHAKIADVQLPQGVWHEDEFYGKSEIYRDRLTLPVTINQASAGATLTVTYQ

: .. :: :. * . *

Sli-DsbD GNKPSGWDKAAEAKVVAPANTSGKDGTTVVIGESKSDH----------------------

Bs-BdbD GNE-----AVSGQPSIKGQPVLGKDDAPVTVVEFG-------------------------

Ec-DsbD GCADAGFCYPPETKTVPLSEVVANNAAPQPVSVPQQEQPTAQLPFSALWALLIGIGIAFT

* . : . .:: :. :

Sli-DsbD ------------------------------------------------------------

Bs-BdbD ------------------------------------------------------------

Ec-DsbD PCVLPMYPLISGIVLGGKQRLSTARALLLTFIYVQGMALTYTALGLVVAAAGLQFQAALQ

Sli-DsbD ---------------------------------------------------VIHLYEDPR

Bs-BdbD ---------------------------------------------------------DYK

Ec-DsbD HPYVLIGLAIVFTLLAMSMFGLFTLQLPSSLQTRLTLMSNRQQGGSPGGVFVMGAIAGLI

.

Sli-DsbD **CPGC**AAMEQSIGETVNKG------------------------------------------

Bs-BdbD **CPSC**KVFNSDIFPKIQKD------------------------------------------

Ec-DsbD **CSPC**TTAPLSAILLYIAQSGNMWLGGGTLYLYALGMGLPLMLITVFGNRLLPKSGPWMEQ

*. * . .

Sli-DsbD -----------------------------MED-GDYKLSFTVGTFLDGNLGGEGSKNALS

Bs-BdbD -----------------------------FIDKGDVKFSFVN-----VMFHGKGSRLAAL

Ec-DsbD VKTAFGFVILALPVFLLERVIGDVWGLRLWSALGVAFFGWAFITSLQAKRGWMRIVQIIL

* :.:.

Sli-DsbD ALGAALNVSPEAFVDYKTALYSTKYHPEESTDEFAKDDYLIKVADS--------------

Bs-BdbD ASEEVWKEDPDSFWAFHEKLFEKQPDTEQ---EWVTPAVLGDLAKS--------------

Ec-DsbD LAAALVSVRPLQDWAFGATHTAQTQTHLNFTQIKTVDELNQALVEAKGKPVMLDLYADWC

. * : : . :..:

Sli-DsbD ----------VDALKGNKKFQDAVEKGTYDAWAMRMSKSFDKAEGVESTPTIKINDKVVE

Bs-BdbD ----------TTKIKP-ETLKDNLDKETFASQVEKDSELNQKMN-IQATPTIYVNDKVIN

Ec-DsbD VACKEFEKYTFSDPQVQKALADTVLLQANVTANDAQDVALLKHLNVLGLPTILFFDGQGQ

: : : * : : : . * : . *** . * :

Sli-DsbD TPSTPDAWQKALKDAGVTK------

Bs-BdbD KFMDYDEIKETIEKELKGK------

Ec-DsbD EHPQARVTGFMDAETFSAHLRDRQP

. :
